# Regulation of Nrf2 by Mitochondrial Reactive Oxygen Species in Physiology and Pathology

**DOI:** 10.3390/biom10020320

**Published:** 2020-02-17

**Authors:** Shuya Kasai, Sunao Shimizu, Yota Tatara, Junsei Mimura, Ken Itoh

**Affiliations:** 1 Department of Stress Response Science, Center for Advanced Medical Research, Hirosaki University Graduate School of Medicine, 5 Zaifu-cho, Hirosaki 036-8562, Japan; kasai-s@hirosaki-u.ac.jp (S.K.); sunao_shimizu@kagome.co.jp (S.S.); jmimura@hirosaki-u.ac.jp (J.M.); 2 Department of Nature & Wellness Research, Innovation Division, Kagome Co., Ltd. Nasushiobara, Tochigi 329-2762, Japan; 3 Department of Glycotechnology, Center for Advanced Medical Research, Hirosaki University Graduate School of Medicine, 5 Zaifu-cho, Hirosaki 036-8562, Japan; ytatara@hirosaki-u.ac.jp

**Keywords:** mitochondrial ROS, Nrf2, Keap1, PGC1α, Klf9, ATF4, Sirt6

## Abstract

Reactive oxygen species (ROS) are byproducts of aerobic respiration and signaling molecules that control various cellular functions. Nrf2 governs the gene expression of endogenous antioxidant synthesis and ROS-eliminating enzymes in response to various electrophilic compounds that inactivate the negative regulator Keap1. Accumulating evidence has shown that mitochondrial ROS (mtROS) activate Nrf2, often mediated by certain protein kinases, and induce the expression of antioxidant genes and genes involved in mitochondrial quality/quantity control. Mild physiological stress, such as caloric restriction and exercise, elicits beneficial effects through a process known as “mitohormesis”. Exercise induces NOX4 expression in the heart, which activates Nrf2 and increases endurance capacity. Mice transiently depleted of SOD2 or overexpressing skeletal muscle-specific UCP1 exhibit Nrf2-mediated antioxidant gene expression and PGC1α-mediated mitochondrial biogenesis. ATF4 activation may induce a transcriptional program that enhances NADPH synthesis in the mitochondria and might cooperate with the Nrf2 antioxidant system. In response to severe oxidative stress, Nrf2 induces Klf9 expression, which represses mtROS-eliminating enzymes to enhance cell death. Nrf2 is inactivated in certain pathological conditions, such as diabetes, but Keap1 down-regulation or mtROS elimination rescues Nrf2 expression and improves the pathology. These reports aid us in understanding the roles of Nrf2 in pathophysiological alterations involving mtROS.

## 1. Introduction

Although reactive oxygen species (ROS) are produced as byproducts of cellular respiration in the mitochondria or cellular metabolisms, cells actively generate them by NADPH oxidases (NOX) and use them as native immune response or cellular signaling molecules [1]. Mitochondria also use ROS as a signal in response to the increased energy demand to change the content and the assembly of mitochondrial electron transport chain (ETC) or to increase mitochondrial biogenesis (i.e., reactive biogenesis) [2]. Furthermore, mitochondrially generated ROS has health-promoting effects known as mitohormesis (discussed later) [3]. For a small amount of ROS to act as signaling molecules in the cells, cellular redox state is maintained with a delicate balance between ROS generation and elimination. In addition to directly oxidizing cellular macromolecules such as DNA, proteins, and lipids, excess ROS cause oxidative stress by depleting glutathione and NADPH as well as disturbing the redox balance to inhibit redox signaling in the cells [4]. Oxidative stress is involved in the pathogenesis of several diseases, such as metabolic syndrome, cancer, and neurodegenerative disease [5]. In certain pathological conditions, ROS is generated by the uncoupling of NO synthase [6] or xanthine oxidase [7] in addition to mitochondria or NOX.

Under oxidative stress conditions, cells remodel metabolism as well as gene expression to maintain redox homeostasis by activating NF-E2-related factor 2 (Nrf2) and other stress response pathways. Nrf2 is known as a master regulator that regulates the transcriptional activation of genes involved in antioxidation, antioxidant biosynthesis and metabolic shift [8]. Under unstressed conditions, Nrf2 is constitutively ubiquitinated by Kelch-like ECH-associated protein 1 (Keap1) and Cullin-3 E3 ligase and degraded by the 26S proteasomal pathway. Keap1-mediated Nrf2 degradation is abrogated by the oxidation of Keap1 [9]. Upon Keap1 binding site saturation with Nrf2, newly synthesized Nrf2 translocates to the nucleus and induces the transcriptional activation of target genes with antioxidant response elements (AREs) in their gene regulatory regions [10]. Thus, Nrf2 maintains redox homeostasis in the cells that enables redox signaling [11].

**Figure 1:**
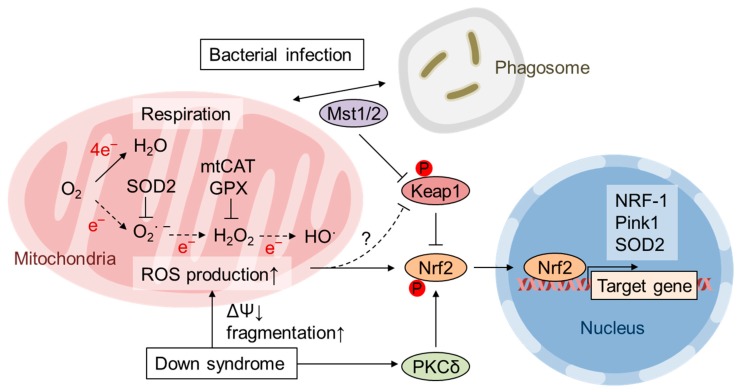
Mitochondrial ROS (mtROS) production and Nrf2 activation by temporal SOD2 depletion, bacterial infection, and Down syndrome. Superoxide (O_2_^∙ −^) produced in the mitochondrial matrix is readily reduced by SOD2 to hydrogen peroxide (H_2_O_2_), which is then reduced by glutathione peroxidase (GPX) to water [12]. Short-term SOD2 depletion in mouse embryos increases mtROS; however, at the postnatal stage, it activates antioxidant defense by Nrf2 and remodels mitochondrial function in the liver [13]. In fibroblasts derived from Down syndrome patients, elevated mtROS production is counteracted by Nrf2, which is activated by PKCδ-mediated phosphorylation [14]. The expression of mitochondrially targeted catalase (mtCAT) alleviates oxidative stress and inhibits Nrf2 activation in fibroblasts from Down syndrome patients [14]. Bacterial infection stimulates macrophages to induce ROS production via NADPH oxidase, and mitochondria are used to eliminate phagocytosed bacteria [15]. Mst1/Mst2 regulates the recruitment of mitochondria to phagosomes and activates Nrf2 by phosphorylating Keap1 to protect the host from excess ROS [15]. Nrf2 induces the expression of cytoplasmic enzymes as well as genes involved in mitochondrial biogenesis and quality control, such as NRF-1, Pink1 and SOD2 [16,17,18,19].

Mitochondria are known as a major source of cellular ROS production; oxidative phosphorylation (OxPhos) converts O_2_ into H_2_O by four-electron reduction, but a small percentage of O_2_ is converted into superoxide anion radicals by one-electron reduction (Figure 1). Superoxide is converted to O_2_ and H_2_O_2_ by superoxide dismutase (SOD). Three different *SOD* genes exist in the eukaryotic genome: *SOD1* encodes cytoplasmic Cu/Zn-SOD, *SOD2* encodes mitochondrial Mn-SOD, and *SOD3* encodes extracellular Cu/Zn-SOD. Superoxide is not thought to be a strongly oxidizing molecule; however, it can inactivate enzymes with iron-sulfur clusters, such as aconitase, and release free iron [12]. Free iron can mediate unfavorable Fenton reactions in which ferrous ions reduce H_2_O_2_ to the most reactive hydroxyl radical. H_2_O_2_ is reduced to H_2_O by glutathione peroxidase (GPX) or peroxiredoxin. Alternatively, H_2_O_2_ is converted to O_2_ and H_2_O by peroxisomal catalase [12] (Figure 1). In the current article, we will discuss the roles of Nrf2 in mitochondrial quality control, focusing on recent studies on Nrf2 regulation by mitochondrial ROS (mtROS) production.

## 2. Nrf2 Contributes to the Homeostasis of Mitochondrial Function

Nrf2 activation occurs during evasion from Keap1-mediated proteasomal degradation in the cytosol or GSK3β-mediated proteasomal degradation in the nucleus [20]. Most typical Nrf2 activators are exogenous electrophilic compounds that preferentially react with the reactive cysteine residues of Keap1, disabling its ubiquitination activity (i.e., the “canonical pathway”) or growth factors that inactivate GSK3β via PI3K activation [10]. As discussed later, p62 is often responsible for the so-called “noncanonical pathway” that activates Nrf2 in response to various cellular stresses independent of Keap1 oxidation [21]. In addition, the phosphorylation of serine 40 of Nrf2 might partially regulate the association of Nrf2 with Keap1, leading to Nrf2 activation [22]. Recently, Suzuki et al. reported that Keap1 acts as a sensor for exogenously added H_2_O_2_ by forming an intramolecular disulfide bond [23]. Keap1 mainly localizes in the perinuclear cytosol suitable to undertake the abovementioned functions [24], although a mitochondrial subpopulation has also been reported [25].

Considering crosstalk with Nrf2 and the mitochondria, how Nrf2 or its target genes affect mitochondrial function is important. Consistent with the function of the Keap1-mediated “canonical” pathway, “canonical” Nrf2 target genes are those that detoxify environmental electrophiles in the cytosol, such as glutathione *S*-transferases or cytosolic antioxidant enzymes, as well as those involved in the synthesis of antioxidants such as glutathione and NADPH, in the cytosol [26]. However, as reviewed by us and others (we are not going to detail in this review), Nrf2 activation leads to mitochondrial biogenesis, mitophagy and an increase in the mitochondrial antioxidant response as well as the activation of OxPhos via the regulation of substrate availability [18,27,28,29]. Although Nrf2 activation is often associated with the induction of mitochondrial localized antioxidant enzymes such as thioredoxin reductase-2 (Txnrd2), peroxiredoxin 3 (Prdx3) and 5 (Prdx5), and SOD2, its regulatory mechanisms are not fully understood [18]. For example, although it has been reported that a GSK3β inhibitor induces the accumulation of both Nrf2 and Txnrd2 in diabetic rat kidneys [30], to the best of our knowledge, the direct regulation of Txnrd2 transcription by Nrf2 remains unknown. Cherry et al. demonstrated that Nrf2 binds to the ARE in the SOD2 promoter during *Staphylococcus aureus*-induced peritonitis in mice [19]. Nrf2 has also been shown to directly bind to the promoter regions of nuclear respiratory factor 1 (NRF-1) and PINK1 [16,17], which are mainly responsible for mitochondrial biogenesis and quality control, respectively. However, it has been reported that Nrf2 induces Prdx3 and Prdx5 expression via NRF-1 [31]. Peroxisome proliferator-activated receptor γ coactivator (PGC) family members, including PGC-1α, PGC-1β, and PGC-related coactivator (PRC), are cofactors that regulate mitochondrial biogenesis via NRF-1 and nuclear respiratory factor 2 (NRF-2). Cherry et al. demonstrated that Nrf2 and PGC-1α physically interact and coregulate SOD2 gene expression in *S. aureus*-induced peritonitis [19].

## 3. Activation of Nrf2 by mtROS

In contrast to exogenous electrophiles and ROS, the mechanism of Nrf2 activation by mitochondrial ROS production may be more complicated considering that superoxide and H_2_O_2_ produced in the mitochondrial matrix do not easily diffuse into the cytoplasm [32] and that generated mitochondrial superoxide is efficiently converted to H_2_O in mitochondria [33]. Generally, various mitochondrial abnormalities can be signaled to the cytosol by non-ROS-related mechanisms, as seen in mitochondrial retrograde signaling, such as in the *C. elegans* ATFS-1 system, in which the drop in membrane potential inhibits peptide transport activity and proteolysis, leading to the nuclear translocation of ATFS-1 [34]. Regarding Nrf2, it has been reported that alterations in complex I activity may determine Nrf2 gene expression via the ERK5-myocyte enhancer factor 2 (MEF2) pathway in a ROS-independent manner [27,35]. However, several reports have shown that changes in mtROS generation through alterations of the level of SOD2 [36] or mitochondria-localized paraquat [37] actually change the cellular response, indicating that alterations in mtROS actually signals to the cytosol using unknown signaling mediators or cascades.

It is known that mtROS activates Nrf2. Oxidation of the extracellular environment by a low cysteine/cystine ratio increases both cellular and mtROS levels and activates Nrf2 in NIH 3T3 mouse embryonic fibroblasts (MEFs) [38]. Although it is unclear how ROS are produced in this system, cellular ROS elevation and Nrf2 activation are suppressed in mitochondria-specific thioredoxin-2 transgenic MEFs, indicating that Nrf2 activation is somehow induced by mtROS [38]. Piantadosi et al. showed that Nrf2 is also involved in carbon monoxide (CO)-induced mitochondrial biogenesis in the heart [16]. CO is endogenously generated by heme oxygenase-1 (HO-1), and HO-1 overexpression stimulates mitochondrial H_2_O_2_ production, which activates the Akt-GSK3β-Nrf2 pathway. Nrf2 activates the transcription of NRF-1 by directly binding to ARE in the NRF-1 promoter. Zamponi et al. demonstrated that Nrf2 activation counteracts ROS-mediated deterioration effects in human fibroblasts from Down syndrome (DS) patients as well as in MEFs from Dp16 mice, a model of DS with segmental trisomy [14]. DS fibroblasts and Dp16 MEFs exhibit an increase in both cytoplasmic and mtROS and impaired mitochondrial membrane potential and fragmentation. DS cells exhibit increased cellular accumulation of Nrf2 and its target gene *HO-1*, and activation of PKCδ by caspase-3-dependent cleavage is responsible for Nrf2 activation via Ser40 phosphorylation. The transgenic expression of catalase fused to a mitochondrial targeting signal (mtCAT) in mice demonstrated that mitochondrial H_2_O_2_ elimination prolongs lifespan and suppresses age-associated diseases, including nonhematopoietic malignancy, decreased cardiac and skeletal muscle function, lipid-induced insulin resistance, and abnormal amyloid β production [39,40,41,42,43]. Similarly, the expression of mtCAT in DS cells reduces both cytoplasmic and mitochondrial ROS and restores mitochondrial dysfunction as well as Nrf2 phosphorylation and accumulation [14] (Figure 1). Nrf2 is activated in macrophages during the immune response to protect them from the marked ROS production required to eliminate pathogens [15]. Geng et al. showed that the kinases Mst1 and Mst2 are necessary for the colocalization of mitochondria and phagosomes so that sufficient ROS are produced for bactericidal activity [44]. Although Mst1/Mst2-deficient macrophages fail to produce ROS in response to Toll-like receptor stimulation, they show increases in basal ROS levels, protein oxidation, DNA damage and apoptosis [15]. Bacterial infection and mitochondrial ROS production induce the translocation of Mst1/Mst2 from the cytosol to mitochondria. Keap1 is also translocated to mitochondria and phosphorylated by Mst1/Mst2 at four Ser/Thr residues in its N-terminus that disable Keap1 dimerization and activate Nrf2 [15] (Figure 1). Thus, these observations suggest that mitochondrial ROS activate Nrf2 mostly by indirect and kinase-dependent mechanisms.

## 4. Role of mtROS and Nrf2 in Mitohormesis

**Figure 2:**
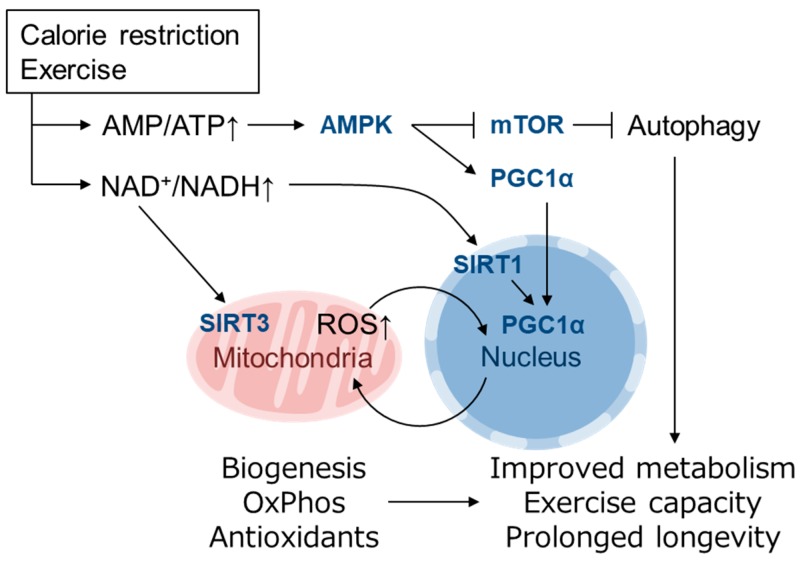
Mitohormesis caused by mild metabolic stress elicits an antioxidant defense response. Metabolic stress, such as caloric restriction and exercise, causes a decline in ATP levels and an increase in the NAD^+^/NADH ratio that is sensed by downstream pathways that promote mitochondrial function and increase ATP synthesis [50,55]. An increase in AMP/ATP activates AMPK, which induces mitochondrial biogenesis via PGC1α activation and enhances autophagy via mTOR inhibition. An increase in NAD^+^/NADH activates the NAD^+^-dependent deacetylases SIRT1 and SIRT3 [61]. Mitochondrially localized SIRT3 activates enzymes involved in mitochondrial ATP synthesis, whereas nuclear SIRT1 also activates PGC1α [50,55]. PGC1α activates the expression of genes involved in mitochondrial biogenesis, fatty acid oxidation, oxidative phosphorylation (OxPhos) and the antioxidant defense system. These adaptations resolve energy shortages and improve metabolic health, exercise capacity and longevity [3].

“Free radical theory” of aging by Harman et al. propose that ROS production and accumulation, which was mainly produced by the disturbed mitochondrial function during aging, causes DNA mutations and degenerative diseases [45]. However, it is becoming clear that small amount of ROS act as signaling molecules in the cells and they elicit health-promoting effects. Reduced glucose metabolism or impaired insulin/IGF signaling alters mitochondrial metabolism to extend life span in various model organisms [3]. MtROS acts as a signaling molecule in these setting to activate beneficial retrograde signaling that regulates mitochondrial dynamics, quality control, proteostasis, biogenesis, and the cellular defense system. In the field of toxicology or radiation medicine, hormesis refers to the preconditioning events in which a subtoxic dose of a substance provokes an endogenous cytoprotective response and confers resistance against later higher amounts of the same toxicants. Patrick Tapia proposed the concept of “mitohormesis” such that weak mitochondrial stress mediates beneficial outcomes of calorie restriction, intermittent fasting, exercise, and dietary phytochemicals with accompanying stoichiometric amount of ROS being essential for the response [46]. The theory was supported by following studies and now multiple pathways are known to mediate the mitohormetic response [3,47]. Mitochondrial stress accompanies not only ROS production and ATP decline, but also accumulation of unfolded protein, decrease in Ca^2+^ buffering, and alteration in metabolite of TCA cycle, OxPhos, fatty acid oxidation, etc. [48]. Ca^2+^ release from mitochondria in muscle during exercise stimulates mitochondrial respiration and biogenesis via calmodulin-dependent protein kinase and calcineurin [49]. An increase in the NAD^+^/NADH and AMP/ATP ratio and a decrease in insulin/IGF signaling or mechanistic target of rapamycin (mTOR) signaling may mediate cellular adaptive responses leading to mitohormesis [3,50]. The NAD^+^-dependent deacetylase Sir2 is reported to promote longevity in yeast [51,52] as well as *C. elegans* and *Drosophila* [53,54]. In mammals, there are 7 Sir2 homolog family members, SIRT1-SIRT7, which have diverse functions in various organelles [50]. Mitochondrial biogenesis induced by a low energy state is mediated by PGC1α, which is a transcriptional cofactor of PPARγ, ERRα, FoxO1 as well as NRF-1 [55,56]. PGC1α is regulated by both transcriptional and posttranslational modifications, the latter of which includes phosphorylation by AMPK (activated by high AMP/ATP ratio) and deacetylation by SIRT1 (activated by high NAD^+^/NADH ratio) [50,55] (Figure 2). Recently, it was reported that PGC1α activity is also regulated by oxygen availability via Lys224 demethylation by the oxygen-dependent demethylase KDM3A [57]. The inhibition of mTOR complex 1 (mTORC1) by AMPK as well as amino acid starvation induces autophagy, which is also involved in longevity in yeast, invertebrates and mice [58]. Mitochondrial biogenesis is also induced by physical exercise [59,60] and has been proposed to extend lifespan, at least in part, by activating the same pathways [3]. Increased NAD^+^/NADH also activates the mitochondrially localized family member SIRT3, which up-regulates ATP synthesis by activating enzymes in the TCA cycle, ETC and fatty acid oxidation and contributes to antioxidant defense by SOD2 induction via FoxO1 deacetylation [61] (Figure 2). Mitochondrial unfolded protein response (UPR^MT^) is also thought as a mitohormetic machinery [62]. In UPR^MT^, expression of mitochondrial chaperones and proteases is up-regulated in response to mtDNA depletion [63] and inhibition of mitochondrial chaperone TRAP1 or protease LONP1 [64]. UPR^MT^ in nematode is mediated by ATFS-1 as described above [34], whereas mammalian UPR^MT^ is thought to be regulated by ATF4, ATF5 and CHOP [65].

In *C. elegans*, skinhead-1 (SKN-1) is the functional homolog of mammalian Nrf1 and Nrf2; however, SKN-1 activation by oxidative stress is mediated by p38 MAPK because Keap1 is absent in *C. elegans* [66]. SKN-1 promotes longevity in wild-type worms and is necessary for longevity extension by multiple pathways, including reduced insulin/IGF-1 signaling (DAF2), mTOR inhibition, and dietary restriction [66]. In contrast, few reports have investigated the role of Nrf2 in the mitohormesis-like response in mammals. Pulliam et al. reported Nrf2 activation in mice lacking surfeit locus protein 1 (*SURF1*), an assembly factor of cytochrome c oxidase (COX/Complex IV) encoded in the nuclear genome [67]. Loss-of-function mutations in the *SURF1* gene lead to mitochondrial disease and Leigh syndrome in humans [68]. *Surf1*-KO mice exhibit decreased COX activity in most tissues and reduced endurance capacity but no neurodegeneration symptoms. Interestingly, *Surf1*-KO mice are resistant to kainic acid-induced neurotoxicity and show a prolonged lifespan [67,69]. *Surf1*-KO mice show a decrease in mitochondrial ROS and an increase in mitochondrial number and PGC1α expression in both heart and skeletal muscle. *Nrf2* and *HO-1* expression is elevated in the heart, whereas the expression of UPR^MT^ genes, *Hsp60*, *ClpP*, and *Lonp1*, is increased in skeletal muscle [67]. Cox et al. revealed that the temporary depletion of SOD2 expression in embryogenesis potentiates Nrf2 signaling in later developmental stages [13]. The authors showed that inducible shRNA-mediated SOD2 knockdown during embryonic days 8.5-12.5 increased oxidative stress hallmarks and reduced aconitase activity without newborn lethality. The adapted mouse livers had restored SOD2 expression and aconitase activity and exhibited decreased mitochondrial respiration, decreased ROS levels, and increased mitochondrial content at 4 weeks old. The gene expression profile identified a gene subset with increased expression in which cis elements of PPARγ, PGC1α and Nrf2 were significantly enriched. These responses were also observed in SOD2-knockdown MEFs. When the down-regulated SOD2 expression was restored after transient SOD2-knockdown, MEFs exhibited increased mitochondrial content, Nrf2 target gene expression induction, and reduced maximal respiration and were resistant to menadione-induced oxidative stress. This adaptive response possibly remodels mitochondrial function so that ROS production is reduced by the increased number of mitochondria and restricted respiration rate as well as increased antioxidant defense response [13]. The molecular mechanisms by which Nrf2 activation is achieved and is maintained for a prolonged period after transient stress stimulation remain to be clarified.

## 5. Role of mtROS and Nrf2 in Mediating the Healthy Effect of Exercise

**Figure 3:**
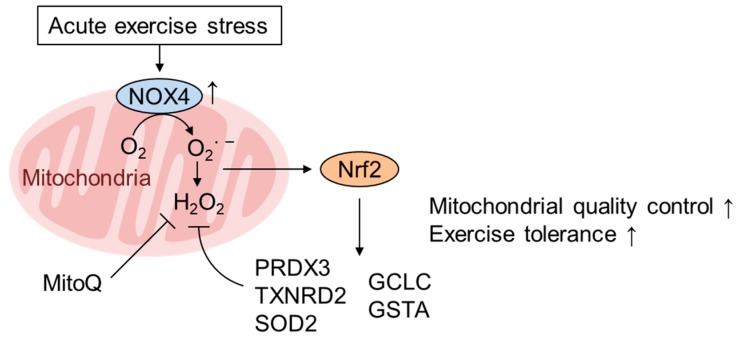
Acute exercise activates Nrf2 via NOX4 expression in the heart. Acute exercise stress (AES) stimulates cardiac performance and exercise capacity via the expression of genes involved in mitochondrial quality control (PRDX3, TXNRD2, and SOD2) as well as cytoplasmic ROS scavenging (GCLC, GSTA1/2) in a NOX4- and Nrf2-dependent manner [73,75]. NOX4 induced by AES localizes to various organelles, including the plasma membrane, nucleus, endoplasmic reticulum (ER), and especially mitochondria, in the heart and produces ROS to activate Nrf2 [78,79,80]. The AES-induced decrease in mtROS is inhibited by Nrf2 deficiency but rescued by the mitochondria-targeted antioxidant MitoQ [73].

Physical exercise activates the cellular response that mimics mitohormesis. Exercise exerts wide-ranging beneficial physiological effects both systemically and locally (i.e., tissue intrinsically) depending on both the strength and duration and confers favorable effects on general wellbeing and various age- and lifestyle-related diseases such as obesity and diabetes [70]. The beneficial effects of exercise partly depend on the improvement in mitochondrial function, including increases in mitochondrial biogenesis and improved respiratory capacity [70]. The beneficial effects of exercise, at least in part, seem to be mediated by ROS signaling [71], as they are mitigated by antioxidants such as vitamin C [72] or suppressed by ROS-metabolizing enzymes [73]; however, ROS appear to derive not from the ETC, but likely from other sources, such as NOX. One of the beneficial effects of exercise is the increase in antioxidant enzymes. Many studies have shown that Nrf2 is activated in response to several modalities of exercise both in cardiac myocytes and skeletal muscle [74]. Here, we discuss the Nrf2 activation mechanisms in these tissues. Muthusamy et al. demonstrated that acute exercise stress (AES) results in the activation of Nrf2/ARE signaling and the subsequent enhancement of antioxidant defense pathways in the mouse heart [75]. They argued that mtROS activates Nrf2 based on the fact that NOX4 and SOD2, but not NOX2 and SOD1, are induced by AES. Although different NOX4 subcellular localization has been reported, including in the nucleus and endoplasmic reticulum [76,77], NOX4 has been shown to mainly localize in mitochondria, especially in the heart [78,79,80]. Indeed, several beneficial effects of exercise in the vasculature and skeletal muscle, such as increased physical activity, are diminished in NOX4-knockout mice [81]. Furthermore, Hancock et al. conclusively demonstrated that Nrf2 activation by AES is mediated by NOX4 and contributes to mitochondrial quality control using cardiomyocyte-specific knockout of NOX4 and Nrf2 (csNrf2 KO) [73]. Both ADP-limited and maximal OxPhos capacity are significantly decreased in csNrf2-KO mice only after exercise. Intriguingly, the expression levels of the mitochondrial antioxidants peroxiredoxin-3, Txnrd2 and SOD2 are increased, and mtROS are decreased by exercise in wild-type hearts but not in csNrf2-KO hearts (Figure 3). Consistently, the mitochondrial defect in the csNrf2-KO heart was rescued by the mitochondria-specific antioxidant mitoquinone (MitoQ), clearly demonstrating that Nrf2 regulates redox status in the heart. Therefore, we surmise that mitochondrial ROS are involved in Nrf2 activation in response to AES in the heart.

In contrast to the heart, muscle has several subtypes of muscle fibers differing in their metabolism, and divergent ROS and/or NO signals may mediate cellular signaling in response to exercise [70]. Merry et al. demonstrated that an acute (1 h) bout of treadmill exercise and subsequent 6 weeks of treadmill exercise training increases the expression of Nrf2 mRNA and its target genes in skeletal muscle in a ROS- and NO-dependent manner [82]. Mitochondrial biogenesis monitored both by mtDNA copy number and citrate synthase activity is increased by treadmill training in an Nrf2-dependent manner via the regulation of PGC1α, NRF-1, and TFAM. Recently, Yamada et al. demonstrated that p62 regulates Nrf2 activation in response to AES specifically in oxidative muscles [83]. Using skeletal muscle-specific p62- and Nrf2-KO mice, the authors demonstrated that acute exercise up-regulates the protein expression of SOD1 and SOD3 in an Nrf2- and p62-dependent manner. p62 is a multifunctional cellular signaling molecule and acts as an autophagy adaptor protein by binding to ubiquitinated proteins and LC3. p62 is also known as an Nrf2 activator [84]. It has an STGE motif, the phosphorylation of which increases the binding affinity for Keap1 by approximately 100-fold. Thus, the phosphorylation of the p62 STGE motif by mTOR activates Nrf2 during selective autophagy [85], and its phosphorylation by Tak1 in response to intestinal flora up-regulates basal Nrf2 expression in colonic epithelial cells [86,87]. We also examined the Nrf2 target genes in the hearts of p62-KO mice and found that the basal expression of Nrf2 target genes such as GSTA1 was repressed without a change in Nrf2 mRNA levels, indicating that p62 regulates basal Nrf2 activation in the heart (our unpublished observations). Thus, it is plausible that ROS and NO that are generated at multiple sites are responsible for Nrf2 activation, but p62 phosphorylation may mediate ROS signaling to activate Nrf2 in oxidative muscles.

## 6. Oxidative Stress Thresholding by Nrf2 and Klf9

**Figure 4:**
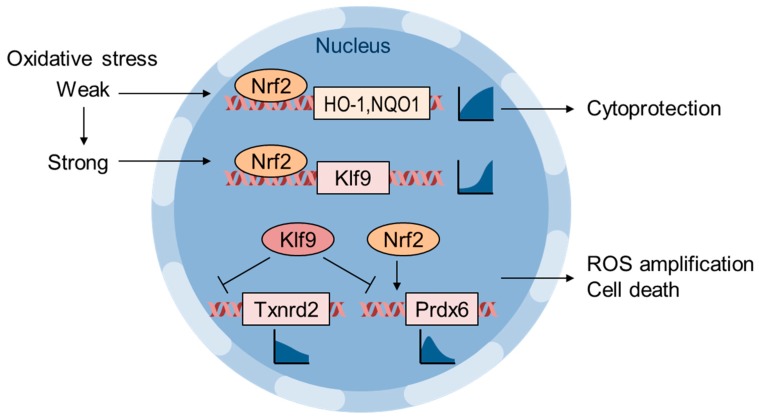
Weak and strong oxidative stresses induce differential gene expression via the Nrf2 and Klf9 axes. Nrf2 induces the expression of cytoprotective genes, such as HO-1 and NQO1, in response to a wide range of oxidative stresses [88]. High-dose H_2_O_2_ or sulforaphane induces Klf9 expression, which represses Txnrd2 gene expression as well as that of other Nrf2-target genes, such as Prdx6, and induces cytotoxic ROS production [88,89]. The line graphs represent the oxidative stress level (*x*-axis) and transcription (*y*-axis) relationship.

Zucker et al. showed that Klf9 is an Nrf2 target gene that is activated in response to lethal oxidative stress and paradoxically increases intracellular ROS via the repression of Txnrd2 [88] (Figure 4). They showed that Nrf2 is recruited to the ARE of HO-1 and NQO1 genes in response to low-dose H_2_O_2_ or sulforaphane, whereas Nrf2 is recruited to the ARE of the Klf9 gene only when cells are exposed to high-dose H_2_O_2_ or sulforaphane. Klf9 overexpression increases basal intracellular ROS as well as H_2_O_2_-induced apoptosis, whereas Klf9 depletion reverses these effects. Txnrd2 overexpression reduces both basal ROS levels and H_2_O_2_-induced cell death in Klf9-expressing cells. Klf9-mediated oxidative stress was evaluated in vivo using bleomycin-induced pulmonary fibrosis model mice, and Klf9-KO mice exhibited a decrease in 8-OHdG levels and fibrosis [88]. Chhunchha et al. also reported an adverse effect of Klf9 in human lens epithelial cells treated with high-dose sulforaphane [89]. Low-dose sulforaphane induces peroxiredoxin-6 (Prdx6) gene expression via Nrf2 and ARE in its regulatory region; however, high-dose sulforaphane suppresses Prdx6 expression via Klf9 and the repressive Klf9 binding element (RKBE). These reports implicate Klf9 as a factor determining cell fate in response to the magnitude of oxidative stress, and Klf9 suppression/inhibition is a possible strategy to alleviate the side effects of Nrf2-activating interventions [89] (Figure 4).

## 7. Pathological Inactivation of Nrf2 by mtROS

MtROS inactivates Nrf2 in certain pathological conditions. Accumulating evidence to date has demonstrated that Nrf2 activity is repressed in various tissues in diabetes patients [90,91,92,93]. However, its precise mechanisms are not currently clear. For factors responsible for Nrf2 repression, one report proposed that BRD4 expression is up-regulated by high glucose concentrations in the mouse podocyte cell line MPC5 [93], whereas another report indicated that GSK3β, which is known to repress Nrf2 activity in a βTrCP-proteasome-dependent manner, is activated in diabetic fetal endothelial cells [94]. BRD4 is a transcriptional cofactor, and high glucose increases Keap1 protein levels in a BRD4-dependent manner in MPC5 cells. Bromodomain and Extraterminal (BET) proteins such as BRD4 have recently been recognized as suppressors of the Nrf2 pathway [95]. These results suggest that multiple factors may contribute to Nrf2 repression in tissue-specific manners. It is of note, however, that in these reports, Nrf2 repression during diabetes or chronic hyperglycemia often accompanies increase of Keap1 protein level. Thus, it is tempting to speculate that Keap1 up-regulation might be an upstream event in Nrf2 repression, and Anzovino et al. demonstrated that Keap1 up-regulation precedes Nrf2 down-regulation in a frataxin-knockout mouse model (discussed below) [96]. In relation to mitochondria, Xiao et al. demonstrated that PINK1/Parkin-mediated mitophagy and Nrf2 down-regulation in diabetic kidney disease are restored by the mitochondria-directed antioxidant MitoQ [97]. MitoQ is a synthetic compound in which ubiquinone (i.e., coenzyme Q10) is tethered to cationic tri-phenyl-phosphonium (TPP). As complex III cannot metabolize MitoQ, it cannot work as an electron carrier in the ETC [91]. Instead, it is believed that its reduced form mitoquinol acts as an antioxidant. Diabetic model db/db mice develop renal tubular injury accompanied by mitochondrial fragmentation, increased cellular and mtROS, and increased apoptosis [97]. Mitochondrial fragmentation can be accounted for by a fusion to fission shift (Drp1 up-regulation and Mfn2 down-regulation) and defects in mitophagy (down-regulation of PINK1, Parkin and LC3II expression) that result in the accumulation of autophagic vacuoles. MitoQ administration normalizes Keap1 up-regulation and Nrf2 down-regulation as well as the abovementioned mitochondrial dysregulation and improved renal pathology and oxidative stress. Human proximal tubular HK-2 cells treated with high glucose concentrations also exhibited mitochondrial dysregulation and increased oxidative stress with impaired Nrf2 and PINK1/parkin signaling. MitoQ treatment reversed these alterations. Furthermore, the down-regulation of ROS production and Drp1 expression and up-regulation of PINK1/Parkin expression by MitoQ are partially dependent on Nrf2 [97]. These results indicate that mtROS that can be neutralized by MitoQ are responsible for Nrf2 repression in diabetes and hyperglycemia.

**Figure 5:**
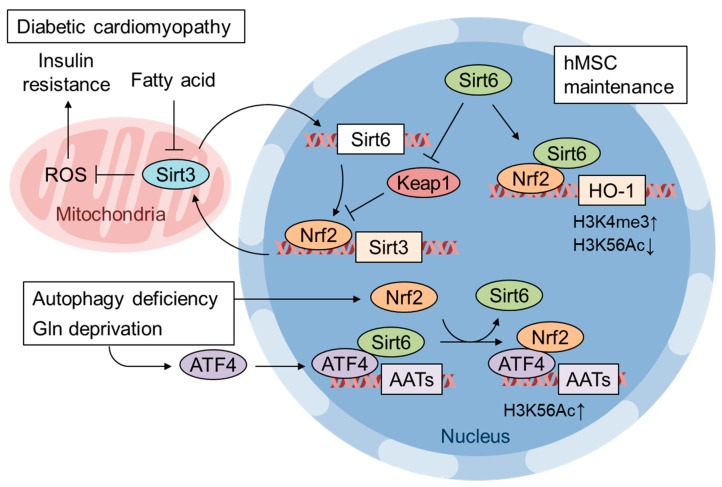
Regulation of gene expression by Nrf2 and Sirt6 crosstalk. Sirt6 expression restores Nrf2 and Sirt3 expression and improves diabetic cardiomyopathy. Sirt6 activates Nrf2 by repressing Keap1 expression as well as interfering with the Nrf2-Keap1 interaction [100]. Sirt6 expression is also important for hMSC maintenance and acts by enhancing Nrf2 target gene expression via epigenetic modifications, increased histone H3 Lys4 trimethylation and decreased Lys56 acetylation [98]. Glutamine starvation in autophagy-deficient cells induces the expression of amino acid transporters (ATTs) to replenish cellular amino acid shortages [106]. Nrf2 is recruited to the ATT gene enhancer by ATF4, where the replacement of Sirt6 with Nrf2 enhances H3K56 acetylation and AAT transcription [106].

Pan et al. identified that SIRT6 and Nrf2 interaction is important for human mesenchymal stem cell (MSC) maintenance [98]. It has been reported that SIRT6 deficiency leads to multiple premature aging events in mice [99]. Additionally, SIRT6-deficient human MSCs exhibit premature senescence, impaired proliferation, and accelerated attrition of the MSC niche when transplanted into nude mice [98]. SIRT6 deficiency increases basal cellular ROS and 8-oxodG levels and sensitizes SIRT6-deficient MSCs to the thioredoxin inhibitor PX-12. RNA sequencing has revealed the suppression of Nrf2 target gene expression, and SIRT6 overexpression rescues gene expression and PX-12-induced apoptosis. A direct interaction between Nrf2 and SIRT6, alterations in epigenetic modifications, increases in H3K4 trimethylation and decreases in H3K56 deacetylation in SIRT6-deficient human MSCs have also been shown [98] (Figure 5). Kanwal et al. showed that Sirt6 transgenic mice are protected from obesity via the activation of Nrf2 [100]. Cardiomyocytes treated with palmitate showed down-regulated SIRT6 expression as well as mitochondrial SIRT3 and increased mitochondrial fragmentation. SIRT3 and SIRT6 positively regulate each other’s expression, and importantly, Nrf2 is also involved in the activation loop. Keap1 mRNA is induced more than 4-fold in palmitate-treated and SIRT6-KD cardiomyocytes. SIRT6 activates Nrf2 by repressing Keap1 transcription and by directly interacting with Nrf2, preventing Keap1 binding and proteasomal degradation. It has been demonstrated that activated Nrf2 induces SIRT3 gene expression via an ARE in its enhancer region (Figure 5). SIRT6 and SIRT3 expression is down-regulated in the hearts of diabetic mice fed a high-fat high-sucrose diet as well as in db/db mice. Mechanistically, Kanwal et al. showed that the reduction in SIRT3 precedes SIRT6 repression during in vitro nutritional overload and that accumulated acetyl CoA acetylates SIRT3 nonenzymatically, leading to the inhibition of SIRT3 activity. Thus, Sirt6 transgenic mice are resistant to diet-induced obesity, cardiac hypertrophy and fibrosis and show improved insulin sensitivity [100]. Collectively, these results show that both chronic hyperglycemia and increased plasma fatty acid levels may contribute to the repression of Nrf2 pathway activity.

Wen et al. found that in addition to diabetes, mtROS-dependent inactivation of Nrf2 occurs in Chagas disease, which is caused by *Trypanosoma cruzi* infection [101]. *Trypanosoma* infection impairs cardiac mitochondrial function, increases mtROS levels, and inactivates Nrf2 function. These alterations are rescued in SOD2-transgenic mice, implying that excess mtROS perturbs proper Nrf2 activation [101]. Friedreich’s ataxia (FRDA) is caused by mutations in the *frataxin* (*FXN*) gene that encodes the mitochondrial matrix protein frataxin, which is important for iron-sulfur cluster biosynthesis in the mitochondria. Indeed, Nrf2 activation by oxidative stress is repressed in fibroblasts derived from FRDA patients or in FXN-knockdown NSC34 neurons [102,103]. The suppressed Nrf2 response in the fibroblasts of FRDA patients is rescued by treatment with a catalase mimetic Euk134, showing that chronic H_2_O_2_ generation causes this response [102]. Also, Nrf2 suppression is observed in dorsal root ganglia (DRG) neurons in the FRDA mouse model and in the striatal muscle-specific KO of FXN [96,104]. Anzovino et al. showed that Keap1 expression is increased and GSK3β is activated in the FXN-KO heart, where pathological alterations are observed, but not in the FNX-KO skeletal muscle [96]. Although direct experimental evidence is lacking, Nrf2 repression might be due to mtROS, as mtROS generation is enhanced in FRDA fibroblasts [105].

## 8. Integrated Stress Response (ISR) Is a Major Downstream Pathway of Mitochondrial Dysfunction

In terms of mitochondrial homeostasis regulation, ATF4 is activated downstream of various mitochondrial perturbations [29]. ATF4 activation by stress signals is mediated by a conserved signaling pathway designated as the integrated stress response (ISR), which is triggered by Ser51 phosphorylation of eukaryotic initiation factor (eIF) 2α by various eIF2α kinases, leading to global translation inhibition and the selective translation of ATF4 [107]. Four stress-responsive eIF2α kinases are conserved in mammals and activated by specific stressors: PKR-like endoplasmic reticulum kinase (PERK) by ER stress, general control nonderepressible-2 (GCN2) by amino acid starvation, heme-regulated inhibitor of translation (HRI) by heme deficiency, and dsRNA-activated protein kinase (PKR) by viral infection [107]. eIF2α phosphorylation attenuates global translation by antagonizing eIF2B guanine nucleotide exchange factor (GEF) activity, which decreases the amount of eIF2 that complexes with GTP and the initiator Met-tRNA (ternary complex) [108]. In contrast, limited ternary complex availability enhances ATF4 translation since two upstream open reading frames (uORFs) in the 5’-UTR region of the mRNA suppress ATF4 ORF translation, which is derepressed by chance when the ternary complex is supplied to the scanning 40S ribosome after the second uORF is skipped [108].

Confining the discussion to mammalian systems, ATF4 activation is likely achieved in tissue- and stress duration- and intensity-dependent manners [109]. Mitochondrial diseases caused by mitochondrial DNA mutations affect specific tissues such as the brain, skeletal muscle, and heart but no other tissues despite systemic cells harboring the same mutation [110]. ATF4 activation has been reported in mitochondrial disease models, such as cybrid cells harboring pathogenic mutations in mitochondrial DNA [111] and Deleter mice, a model of progressive external ophthalmoplegia (PEO) caused by a mutation in the mtDNA helicase Twinkle that leads to mtDNA deletion [112] (other reports are summarized in a previous review [29]). Although some mitochondrial stress, such as OxPhos inhibition and mitochondrial depolarization, induce mtROS elevation and ATF4 activation, other mitochondrial stress, such as the inhibition of mitochondrial translation by doxycycline, activates ATF4 with no alterations in mtROS levels [113].

The involvement of mtROS in ATF4 activation has been demonstrated in limited reports as follows. Kim et al. showed that metformin, an antihyperglycemic agent known to inhibit mitochondrial complex I, increases mtROS and induces FGF21 expression via the PERK-eIF2α-ATF4 pathway, which is suppressed by the mitochondria-targeted superoxide scavenger Mito-TEMPO in rat hepatoma FaO cells [114]. Wang et al. showed that cisplatin resistance in gastric cancer cells is conferred by xCT induction via the GCN2-eIF2α-ATF4 pathway [115]. The ATP synthase inhibitor oligomycin increases both cellular and mitochondrial ROS and activates GCN2, which is blocked by N-acetyl cysteine. This observation is consistent with a report in *C. elegans* in which in vivo GCN2 activation is inhibited by antioxidant treatment [116].

Guo et al. recently clarified that as an alternative pathway of mitochondrial stress-induced ATF4 activation, oligomycin-induced mitochondrial stress is sensed by the mitochondrial protease OMA1 and its substrate DELE1, which activates the HRI pathway without oxidative stress or heme deprivation in human HEK293T cells [117]. Under mitochondrial stress conditions, DELE1 is cleaved by OMA1 in the inner mitochondrial membrane, and its C-terminal portion translocates to the cytoplasm to activate HRI directly. OMA1 is also known to regulate mitochondrial quality by processing OPA1, which accelerates mitochondrial fission under mitochondrial depolarization conditions [118]; therefore, OMA1 can activate intramitochondrial and mitonuclear retrograde signaling to ensure mitochondrial homeostasis.

## 9. Nrf2 and ATF4 Crosstalk in Mitochondrial Retrograde Signaling

DeNicola et al. showed that Nrf2 activation in non-small-cell lung cancer (NSCLC) cells induces the expression of genes involved in the glycine/serine synthesis pathway in an ATF4-dependent manner [119]. The depletion of Nrf2, ATF4 or phosphoglycerate dehydrogenase (PHGDH, the first rate-limiting enzyme in serine biosynthesis) results in the down-regulation of glutathione, the NADPH/NADP+ ratio and nucleotide synthesis as well as anchorage-independent colony formation [119]. The transcriptional activation of ATF4 by Nrf2 has also been reported in human umbilical vein endothelial cells (HUVEC) and in retinal pigment epithelial ARPE-19/HPV-16 cells [120,121].

**Figure 6:**
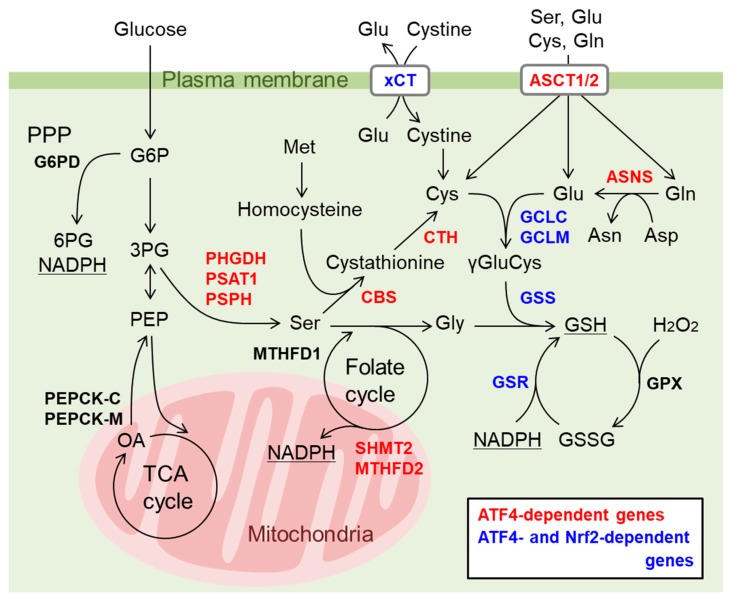
Cooperative regulation of genes involved in glutathione (GSH) and NADPH synthesis by Nrf2 and ATF4. Carnosic acid activates both Nrf2 and ATF4 and induces antioxidant genes and genes involved in amino acid synthesis/transport and GSH synthesis. The contributions of Nrf2 and ATF4 to the gene activation were analyzed by knockdown of Nrf2 or ATF4 and shown with color characters ([124] and our unpublished observation). Induction of cystine transporter xCT and GSH synthetic enzymes (GCLC, GCLM, GSS, and GSR) was dependent on both Nrf2 and ATF4 (genes indicated with blue). Genes involved in serine synthesis (PHGDH, PSAT1, and PSPH), transsulfuration (CBS and CTH), folate cycle (SHMT2 and MTHFD2), and asparagine synthase (ASNS) and Ala/Ser/Cys/Thr transporter-1 and -2 (ASCT1/2) are dependent on only ATF4 and indicated with red. Colored genes are also up-regulated in HSA-UCP1-Tg mice [132] except for ASCT1 and additionally include G6PD, PEPCK-C, PEPCK-M and MTHFD1 (indicted with black). G6PD is a rate-limiting enzyme of pentose phosphate pathway (PPP) that supplies NADPH and is known as an Nrf2 target gene [28]. PEPCKs may redirect TCA cycle metabolites to Ser synthetic pathway and are reported as ATF4-regulated genes [135,136]. Folate cycle converts Ser to Gly and up-regulation of mitochondrial SHMT2 and MTHFD2 may increase mitochondrial NADPH [29].

In addition to the role of ATF4 as a downstream effector of Nrf2 in cancer cells, it is increasingly reported that the direct interaction of Nrf2 and ATF4 is involved in gene expression. He et al. first reported that Nrf2 physically binds to ATF4 in a yeast two-hybrid screen and that the ATF4-Nrf2 heterodimer binds to AREs, further showing the involvement of ATF4 in cadmium-induced *HO-1* expression in a cell type-specific manner [122]. We have also reported that proteasome inhibitors activate both Nrf2 and ATF4 and cooperatively induce the expression of the cystine transporter *xCT* in bladder cancer cells via Nrf2 binding to ARE and ATF4 binding to amino acid response elements (AAREs), respectively [123]. In addition, we recently identified several antioxidant and cytoprotective genes including nerve growth facts (NGF) are cooperatively regulated by both Nrf2 and ATF4 when both are activated by the phytochemical carnosic acid in the human glioma U373MG cells [124]. Interestingly, genes involved in glutathione synthesis are regulated to various extent in either an Nrf2 and ATF4-dependent or in a both Nrf2- and ATF4-dependent manner (Figure 6) ([124] and our unpublished observation). Furthermore, genes involved in NADPH synthesis in the mitochondria such as SHMT2 and MTHFD2 are regulated by ATF4 [29] and those in the cytosol (i.e. pentose phosphate pathway (PPP) and malic enzyme 1) are regulated by Nrf2 [8]. Thus, we surmised that Nrf2 and ATF4 may cooperatively work in the redox homeostasis regulation in the cells. In contrast, the expression of *CHOP* induced by ATF4 is negatively regulated by Nrf2 in thyroid cancer cell lines treated with proteasome inhibitors [125]. The recruitment of ATF4 to the AARE of the *CHOP* gene promoter is attenuated by Nrf2, although neither the ARE nor DNA binding of Nrf2 was observed [125]. Since CHOP is a transcription factor that induces apoptosis [126], Nrf2 activation might be a potential strategy to prevent various stress-induced degenerative diseases by enhancing cytoprotective gene expression and attenuating apoptotic gene expression. Zhang et al. showed that autophagy-deficient cancer cells induce amino acid transporter (AAT) expression in response to glutamine starvation, and the induction is dependent on both ATF4 and Nrf2 [106] (Figure 5). The authors showed Nrf2 expression is increased in autophagy-deficient cells whereas ATF4 expression is induced by glutamine starvation. In this case, Nrf2 knockdown did not affect ATF4 gene induction. In wild-type cells, ATF4 and SIRT6 are recruited to the promoters of AAT genes in response to glutamine starvation. Autophagy deficiency increases the ATF4 and Nrf2 interaction, which suppresses SIRT6 recruitment to the AAT gene promoter and increases H3 Lys56 acetylation, which enhances AAT expression and extracellular amino acid uptake (Figure 5). Although it is unclear how Nrf2 expression is increased in autophagy-deficient cells, the authors pointed out that p62 accumulation by autophagy deficiency may activate Nrf2 as described above [106].

It has been reported that genetic OxPhos uncoupling activates both Nrf2 and ATF4 [127]. UCP1 expression permeates protons through the mitochondrial inner membrane, thereby uncoupling OxPhos to provide a proton gradient for thermogenesis. Skeletal muscle-specific UCP1 transgenic (HSA-UCP1-Tg) mice exhibit a healthy aging phenotype, such as decreased atherosclerosis and obesity and prolonged life span [128,129,130,131]. HSA-UCP1-Tg mice show a decrease in skeletal muscle mass, particularly in fast/glycolytic muscles, compared to slow/oxidative muscles, which is accompanied by an increase in food intake, oxygen consumption and glucose uptake in skeletal muscle [130]. UCP1 overexpression also increases catalase and SOD activity as well as glutathione content in skeletal muscle [130,131]. Ost et al. revealed that the expression of genes involved in spermidine synthesis, serine/glycine synthesis and glutathione synthesis is increased in HSA-UCP1-Tg mice [132] (Figure 6). Spermidine is a polyamine that induces autophagy, confers resistance to oxidative stress, and extends longevity. Coleman et al. demonstrated that elevated NQO1 and catalase activity in HSA-UCP1-Tg mice are abolished in Nrf2-knockout mice [127]. In contrast, Nrf2 deficiency does not affect other phenotypes of the mice, such as a decreased quadriceps mass and fiber size, improvement of metabolic hallmarks in plasma (decreased insulin, diacylglyceride, fatty acids and cholesterol), and increased FGF21 and GDF15 secretion. FGF21 and GDF15 are known as mitokines, hormone whose expression and secretion are induced by mitochondrial stress and improve metabolic health and extend lifespan [133]. HSA-UCP1-Tg mice exhibit an increase in respiration leak consistent with UCP1 function, which is not affected by Nrf2 ablation. In contrast, Nrf2 deficiency lowers the maximum coupled respiration in wild-type and Tg mice but does not alter the expression of respiratory chain complexes [127], probably due to impaired substrate availability, as reported previously [134]. Interestingly, PGC1α expression is elevated in HSA-UCP1-Tg mice and is partially suppressed in Nrf2-knockout mice. Nrf2 activation might induce PGC1α expression in this model, although PGC1α activation by AMPK and Sirt1 rather than transcriptional induction has been reported in another study of HSA-UCP1-Tg mice [131]. Nrf2 deficiency partially decreases ATF4 mRNA and increases BIP expression; however, the activation of the serine/glycine synthesis pathway is independent of Nrf2 [127]. In contrast, *GPX1* expression and total GPX activity are further increased by Nrf2 deficiency, implying that an alternative pathway may compensate for Nrf2 deficiency. The methylglyoxal adduct is decreased in Tg mouse muscle and not significantly affected by Nrf2 deficiency [127]. Taken together, these results identified both Nrf2-dependent and Nrf2-independent pathways in response to UCP1 overexpression in the skeletal muscle; however, their activation mechanisms and biological impact on Nrf2 remain to be understood in future studies.

## 10. Conclusions

In this review, we discussed that Nrf2 is activated on some occasions while inactivated in others by mtROS. It is plausible that high mtROS levels inactivate the signaling cascade that activate Nrf2 leading to the inhibition of Nrf2 activity. However, the different locations of ROS generation (matrix or intermembrane space) or difference of reactive species generated (hydrogen peroxide, superoxide or secondary generation of more complex reactive species such as peroxynitrite, etc.) may also be involved in the Nrf2 inhibition. Thus, further studies are required to clarify these issues. Some evidence support hydrogen peroxide as the responsible downstream ROS as Nrf2 activation is blocked by the expression of mtCAT. Nrf2 and other factors (e.g., sirtuins, ATF4 and Klf9) modulate each other’s function and affect cell fate to recover or undergo cell death. The activation of both Nrf2 and ATF4 exerts remodeling of cellular metabolism that up-regulates glutathione and NADPH to maintain cytoplasmic and mitochondrial redox homeostasis. Antioxidant administration is not always beneficial for health promotion by exercise (discussed in the current review) or cancer chemoprevention [137], probably due to the disturbance of mitohormesis (known as the “antioxidant paradox”). The elucidation of Nrf2-centered network status and the associated molecular mechanisms under various pathophysiological conditions is necessary for understanding healthy homeostasis and predicting the outcomes of Nrf2-activating interventions in the future.

## Abbreviations

AES: acute exercise stress; AMPK: AMP-activated protein kinase; ARE: antioxidant response elements; ASCT: Ala/Ser/Cys/Thr transporter; ASNS: asparagine synthase; ATF4: activating transcription factor 4; ATP: adenosine triphosphate; CBS: cystathionine beta-synthase; CHOP: CCAAT/enhancer-binding protein homologous protein; COX: cytochrome c oxidase; CTH: cystathionine gamma-lyase; DS: Down syndrome; ETC: electron transport chain; G6PD: glucose-6-phosphate dehydrogenase; GCL: glutamate-cysteine ligase; GPX: glutathione peroxidase; GSR: glutathione S-reductase; GSS: glutathione synthetase; GTP: guanosine triphosphate; HO-1: heme oxygenase-1; Keap1: Kelch-like ECH-associated protein 1; Klf9: Kruppel-like factor 9; HSA: human skeletal actin promoter; MEF: mouse embryonic fibroblasts; MSC: mesenchymal stem cell; mtCAT: mitochondrial targeted catalase; MTHFD: methylenetetrahydrofolate dehydrogenase; mtROS: mitochondrial ROS; NADPH: nicotinamide adenine dinucleotide phosphate; NOX4: NADPH oxidase 4; Nrf2: NF-E2-related factor 2; NRF-1: nuclear respiratory factor 1; OxPhos: oxidative phosphorylation; PEPCK: phosphoenolpyruvate carboxykinase; PGC1α: peroxisome proliferator-activated receptor γ coactivator 1α; PHGDH: D-3-phosphoglycerate dehydrogenase; PPP: pentose phosphate pathway; PSAT1: phosphoserine aminotransferase 1; PSPH: phosphoserine phosphatase; Prdx: peroxiredoxin; ROS: reactive oxygen species; SHMT2: serine hydroxymethyltransferase 2; SKN-1: skinhead-1; SOD: superoxide dismutase; SURF1: surfeit locus protein 1; TCA: tricarboxylic acid; Tg: transgenic; Txnrd: thioredoxin reductase; UCP1: uncoupling protein 1; UPR^MT^: mitochondrial unfolded protein response.
